# Correlation of risk factors for ischemic stroke with uric acid/creatinine ratio and cerebral vascular hemodynamic index (CVHI) in physical examination population

**DOI:** 10.12669/pjms.41.3.9912

**Published:** 2025-03

**Authors:** Xue Bai, Hui Wang, Jiangzhe Li, Jinjin Xu, Pan Cai

**Affiliations:** 1 Xue Bai Department of Physical Examination, Baoding No.1 Central Hospital, Baoding 071000, Hebei, China; 2 Hui Wang Department of Urological, Baoding No.1 Central Hospital, Baoding 071000, Hebei, China; 3 Jiangzhe Li Western Medicine Pharmacy Static Dispensing Center, Baoding No.1 Central Hospital, Baoding 071000, Hebei, China; 4 Jinjin Xu Department of Geriatric, Baoding No.1 Central Hospital, Baoding 071000, Hebei, China; 5 Pan Cai Department of Rheumatology and Immunology, Baoding No.1 Central Hospital, Baoding 071000, Hebei, China

**Keywords:** CVHI, Ischemic stroke, Physical examination, Risk factor analysis, Uric acid/creatinine ratio

## Abstract

**Objective:**

To investigate the risk of ischemic stroke, uric acid/creatinine ratio, cerebral vascular hemodynamic index (CVHI) and risk factors of stroke in physical examination population.

**Methods:**

This was a retrospective study. Four hundred cases undergoing physical examination at physical examination center in Baoding No.1 Central Hospital from January 2023 to December 2023 were selected as subjects, and their general data were collected. They were divided into low ratio group, medium ratio group and high ratio group depending on the uric acid/creatinine ratio, and were classified as high-risk group, medium-risk group and low-risk group as per CVHI, the general data of all subjects were compared and analyzed.

**Results:**

Multifactorial Logistic regression analysis was performed after assignment of stroke risk factors as dependent variables yielding that advanced age, smoking, alcohol abuse, hypertension, diabetes mellitus, BMI, hyperlipidemia, and hyperuricemia were risk factors for ischemic stroke(*P*<0.05). Those with high uric acid/creatinine ratio were markedly higher than those with medium and low ratio in the proportion of alcohol abuse, hypertension, diabetes mellitus, and hyperlipidemia, with statistically significant differences (*P*<0.05). Obesity, advanced age, smoking, hypertension, diabetes mellitus, and hyperlipidemia were obviously higher in the high-risk group for stroke than in the medium- and low-risk groups, with statistically significant differences (*P*<0.05).

**Conclusion:**

Risk factors for ischemic stroke predispose to older age groups, and are linked to underlying medical conditions, especially in those with high uric acid/creatinine ratios and those at high risk of stroke. The SUA/Cr ratio is negatively correlated with the CVHI score.

## INTRODUCTION

Recent years have witnessed an increasing standard of living and an accelerating aging process, with the incidence of chronic diseases rising to more than 20% of the total population. These chronic diseases mainly include diabetes, hyperlipidemia and hypertension.^1^ Chronic diseases, a major factor causing cardiovascular and cerebrovascular diseases, impose a heavy burden on people’s health, society and family.^2^ Stroke is an end-involved disease caused by long-term mixed and complex effects of chronic diseases, with an acute onset and rapid progress.

It is a serious non-communicable disease that threatens people’s health, resulting in damage to brain tissue due to interruption of blood flow caused by cerebral vascular rupture or thrombosis. A sustained upward trend in the incidence and mortality of stroke in the population in recent years, and a gradual rejuvenation of the incidence of stroke. Despite the increasing health awareness and the widespread implementation of some chronic disease interventions and control measures, the risk factors for stroke have not been adequately explored, and their control is still unsatisfactory.

Serum uric acid/creatinine (SUA/Cr) ratio is associated with a variety of adverse outcomes, especially atherosclerotic cardiovascular disease. Wang et al.^3^ revealed a certain correlation between SUA/Cr and chronic diseases such as stroke, metabolic syndrome, chronic obstructive pulmonary disease, and osteoporosis. Given this, the present study was designed to explore the risk level of stroke and exposure to risk factors in this population, and to analyze the correlation between SUA/Cr and CVHI, with a view to providing a scientific basis for the development of safe and efficient interventions and preventive measures for chronic stroke.

## METHODS

This was a retrospective study. Four hundred cases undergoing physical examination at physical examination center in Baoding No.1 Central Hospital from January 2023 to December 2023 were selected as subjects. Data were retrieved from our hospital information and management system, and their general data were collected. They were divided into low ratio group, medium ratio group and high ratio group depending on the uric acid/creatinine ratio, and were classified as high-risk group, medium-risk group and low-risk group as per CVHI. The general data of the subjects, such as gender, age, smoking, alcohol consumption, hypertension, diabetes mellitus, hyperuricemia, and hyperlipemia, were analyzed to explore the risk factors associated with the occurrence of ischemic stroke in healthy physical examination population. Meanwhile, the exposure of stroke-related risk factors in different SUA/Cr groups and CVHI was analyzed, and a correlation analysis between SUA/Cr and CVHI was performed.

### Ethical Approval:

The study was approved by the Institutional Ethics Committee of Baoding No.1 Central Hospital (No.: 2023103; Date: October 23, 2023), and written informed consent was obtained from all participants.

### Inclusion criteria:

- Patients aged ≥35 years.
- Local permanent residents (≥2 years of residence in the area of physical examination).
- Those who voluntarily underwent cerebral hemodynamic monitoring, serum uric acid, creatinine and related laboratory tests, and screening of high-risk groups for stroke.

### Exclusion criteria:

- Patients with incomplete demographic and laboratory data.
- Those with the exception of pre-existing chronic diseases, such as chronic kidney disease, and malignant tumors.
- Those who were mentally ill or otherwise unable to cooperate with the study.
- Those with abnormal immune function or chronic inflammatory diseases.
- Those with communication barriers.

Collected their general data, including age, gender, past history, smoking history and drinking history, were collected. Height, body weight, blood pressure, and body mass index of all subjects were measured, and biochemical indexes such as lipids, glucose uric acid, creatinine, and CVHI scores were measured. Based on the uric acid/creatinine ratio, the subjects were categorized into a low ratio group (Group L, SUA/Cr<4), a medium ratio group (Group M, 4<SUA/Cr<5.5), and a high ratio group (Group H, SUA/Cr>5.5), and the general demographic data of the three groups is shown in Table-I.

**Table-I:** Comparative analysis of general data of the three groups (*χ̅±S*).

Indexes	Group L	Group M	Group H	c^2^	P
n	160	148	92		
Gender				0.17	0.62
Male	83	75	49		
Female	77	73	43		
Ethnicity				0.14	0.73
Han	142	125	80		
Other	18	23	12		
Age*				5.68	0.00
35~60	78	55	29		
61~75	43	46	27		
>75	39	47	36		

* P<0.05

### Judgment criteria for general indicators:

***Hypertension:*** systolic blood pressure ≥140 mmHg, and/or systolic blood pressure ≥90 mmHg.^4^

***Hyperlipidemia:*** triglyceride ≥1.7 mmol/L, total cholesterol ≥6.22 mmol/L, low-density lipoprotein cholesterol ≥4.14 mmol/L, or high-density lipoprotein cholesterol (HDL-C) <1.04 mmol/L or ≥1.55^5^.

***Abnormal blood glucose:*** fasting blood glucose ≥7.0 mmol/L or postprandial blood glucose ≥11.1 mmol/L or the level of diabetic patients^6^; (4) High uric acid: uric acid >420 mmol/L for males and >360 mmol/L for females.^7^ (5) Obesity: BMI 18.5–24 kg/m^2^ for normal, BMI 24–28 kg/m^2^ for overweight, and BMI≥28 kg/m^2^ for obesity.

***Judgment criteria for cerebral vascular hemodynamic index (CVHI) scores:*** CVHI scores were determined using a CVHI tester with a probe frequency of 5MHz. The subjects were placed in the supine position to fully expose their necks, and 10 pairs of parameters were examined, including left and right side mean blood flow volume (Qmean), maximum flow velocity (Vmax), minimum flow velocity (Vmin), mean flow velocity (Vmean), peripheral resistance (Rv), characteristic impedance (Zcv), pulse wave velocity (Wv), dynamic resistance (DR), critical pressure (Cp), and the difference between diastolic blood pressure and critical blood pressure (Dp). The above 10 pairs of indexes were calculated as points out of 100; ≥75 points were considered normal, and <75 points were defined as abnormal CVHI, of which 0–24 points were considered as high risk of stroke, 25-49 points as medium risk of stroke, and 50–74 points as low risk of stroke.^8^

### Statistical analysis:

All data were statistically analyzed using the SPSS 26.0 software (SPSS Inc., Chicago, IL, USA). Normally distributed measures were expressed as (*X̅*±*S*), and ANOVA was used for comparison between multiple groups. Independent sample t-test was used between groups, and χ^2^ was used for comparison of rates. Correlates were analyzed using logistic regression analysis, whereas correlations between the SUA/Cr ratio and the CVHI score were analyzed using Spearman’s rank correlation analysis. A P-value <0.05 indicated a statistically significant difference.

## RESULTS

Multifactorial Logistic regression analysis was performed after assignment of stroke risk factors as dependent variables, with α _in_=0.05 and α _out_=0.10. The results concluded that advanced age, smoking, alcohol abuse, hypertension, diabetes mellitus, BMI, hyperlipidemia, and hyperuricemia were risk factors for ischemic stroke (*P*<0.05) (Table-II).

**Table-II:** Logistic regression analysis of risk factors affecting ischemic stroke in healthy physical examination (*χ̅±S*) n=400.

Indexes	β	SE	Waldc^2^	P	OR	95.0%*CI*
Age	3.683	0.427	4.820	0.001	3.308	3.027~3.574
Smoking	3.407	0.433	3.571	0.001	3.262	2.908~3.410
Alcohol abuse	4.863	0.517	4.608	0.002	3.524	3.207~3.611
Hypertension	3.571	0.394	3.508	0.001	3.301	3.131~3.479
Diabetes mellitus	4.017	0.570	5.127	0.000	4.638	3.779~4.803
BMI	3.271	0.382	3.007	0.003	3.125	2.970~3.418
Hyperlipidemia	3.460	0.402	3.617	0.002	3.208	2.875~3.431
Hyperuricemia	3.372	0.418	3.163	0.004	3.072	2.631~3.310

*P<0.05

**Table-III:** Detection of different uric acid/creatinine ratios in different population groups (*χ̅±S*).

Indexer	Group L	Group M	Group H	c^2^	P
n	160	148	92		
Alcohol abuse	11 (7%)	17 (11%)	20 (22%)	4.42	0.03
Hypertension	25 (16%)	32 (22%)	34 (37%)	5.41	0.02
Diabetes mellitus	17 (11%)	20 (14%)	22 (24%)	4.52	0.02
Hyperlipidemia	11 (7%)	15 (10%)	31 (34%)	15.70	0.00

P<0.05.

**Table-IV:** Detection of stroke risk factors in different population groups (*χ̅±S*).

Indexes	Low-risk group	Medium risk group	High-risk group	c^2^	P
n	170	130	100		
Obesity	22 (13%)	18 (14%)	30 (30%)	7.46	0.00
Advanced age	37 (22%)	30 (23%)	41 (41%)	8.37	0.00
Smoking	48 (28%)	45 (35%)	47 (47%)	4.68	0.03
Hypertension	16 (9%)	24 (18%)	30 (30%)	7.62	0.00
Diabetes mellitus	12 (7%)	17 (13%)	21 (21%)	6.14	0.03
Hyperlipidemia	11 (6%)	15 (12%)	23 (23%)	5.10	0.02

*P<0.05.

**Table-V:** Correlation analysis of uric acid/creatinine ratio and stroke risk factors (r).

Stroke risk factors	Group L	Group M	Group H*
Low risk	0.18	0.13	-0.23
Medium risk	0.20	0.12	-0.26
High risk	0.21	0.15	-0.30
*r*	0.57	0.79	-3.47
*p*	0.48	0.25	0.00

* P<0.05.

Based on the detection of different uric acid/creatinine ratios in different population groups, those with high uric acid/creatinine ratio were markedly higher than those with medium and low ratio in the proportion of alcohol abuse, hypertension, diabetes mellitus, and hyperlipidemia, with statistically significant differences (*P*<0.05).

Based on the detection of stroke risk factors (CVHI) in different groups, obesity, advanced age, smoking, hypertension, diabetes mellitus, and hyperlipidemia were obviously higher in the high-risk group than in the medium- and low-risk groups, with statistically significant differences (*P*<0.05). The correlation analysis between SUA/Cr and CVHI suggested that Group-L and Group-M presented no correlation with CVHI (p>0.05), while Group-H presented negative correlation with CVHI (*r*=-3.47, *P*=0.00).

## DISCUSSION

It was shown in our study that advanced age, smoking, alcohol abuse, hypertension, diabetes mellitus, and hyperlipidemia were risk factors for ischemic stroke (*P*<0.05), which is basically in agreement with the findings of Chen et al.^9^ Chronic diseases are heavy pressure causative factors that induce stroke in older adults, and dietary and lifestyle factors are increasing in their share. These risk factors greatly add to the risk of stroke onset when combined over a long period of time.^10^

Our results suggest a high risk of stroke in the elderly as well as in those who smoke, abuse alcohol, have hypertension, hyperlipidemia and hyperuricemia. The elderly themselves suffer from systemic functional attenuation and vascular fragility. If they smoke for a long time, they will suffer further damage to the vascular endothelium under the influence of nicotine stimulation of endothelial cell metabolism. Coupled with long-term physical inactivity, overweight or obesity, metabolites in their bodies will increase in quantity and be discharged slowly, and incomplete oxidation of fats results in the continued accumulation of unsaturated fatty acids and other hazardous substances, which will cause tiny damage in multiple regions. Long-term tiny damage would keep eroding the level of human health.^11^

Studies have shown^12^ that hypertension and hyperlipidemia are the predominant risk factors for stroke in the elderly, and their prevention and intervention are essential for controlling the onset of stroke. Hypertension is such an influential risk factor for the onset of chronic diseases that long-term elevation of blood pressure increases the incidence of coronary heart disease and stroke in the population.^13^ This proves the importance of blood pressure control in the onset of stroke. Furthermore, the increasing number of dyslipidemias indicates an improvement in the nutritional status of the population. However, confined to the awareness level, the majority of residents live in a variety of bad behavior and eating habits. These can be gradually improved with increasing levels of education and its popularization.

A study^14^ confirmed that effective control of stroke risk factors prevents the occurrence and recurrence of cerebrovascular disease efficiently. The Guidelines for the Primary Prevention of Stroke by the American College of Cardiology/American Heart Association divide stroke risk factors into three major categories. The first category is non-modifiable factors, such as age, gender, family history; the second category is controllable factors, such as, BMI, blood glucose, blood lipids and blood pressure, cardiovascular diseases; and the third category is intervenable factors, such as metabolic syndrome, high homocysteine. The second and third categories of factors are preventable and controllable, and at least 90% of stroke patients can be prevented with health management and health intervention. In the context of the popularization of health physical examination, stroke monitoring and post-physical examination health management of the population should be actively carried out, which is of great significance in reducing the risk of stroke and will become a key pathway for disease prevention.

We stratified the SUA/Cr ratio and concluded that those with high ratio were markedly higher than those with medium and low ratio in the proportion of alcohol abuse, hypertension, diabetes mellitus, and hyperlipidemia, with statistically significant differences (*P*<0.05). The cause of such may be related to several factors. First, serum creatinine (Cr) is considered as an indicator for renal evaluation, but it is affected by several factors. SUA is the end product of purine nucleotide catabolism. Normalized SUA/Cr in renal function has been shown to be a modifiable risk factor for cardiovascular disease.^15^

Abnormalities of lipid and glucose metabolism in the human body are proven to interact and influence with purine metabolism disorders. Secondly, studies have demonstrated an association between high levels of SUA and obesity, blood pressure, dyslipidemia, and also diabetes mellitus.^16^ It has been noted that higher SUA levels may be associated with an increased risk of stroke morbidity and mortality in large prospective cohort studies with long-term follow-up in many different countries.^17^ Daily dietary intake, exercise and BMI affect SUA levels directly or indirectly. Caimi et al.^18^ stated that serum uric acid and serum uric acid/creatinine ratio have been shown to be one of the risk factors for the onset of stroke in addition to correlating with whole blood viscosity, plasma viscosity, and plasma viscosity in subjects with subclinical carotid atherosclerosis. Therefore, the SUA/Cr ratio can be used as one of the effective observations for further prevention of stroke onset.

The CVHI score is considered a valuable indicator for risk assessment of first stroke.^19^ The present study showed that the mean and abnormality rates of the CVHI score were negatively correlated with SUA/Cr (*r*=-3.47, *P*=0.00), i.e., the higher the SUA/Cr ratio, the more pronounced was the decrease in abnormality rates and the mean of the CVHI score. The reason for this may be that subclinical arterial stiffness may already exist in healthy individuals, and SUA levels may contribute to the prediction of atherosclerosis.

The close association of hemodynamic abnormalities with the onset and progression of atherosclerosis. More studies by Yamashiro and colleagues^20^ have shown that individuals with atherosclerotic cerebrovascular disease and significant hemodynamic abnormalities are at a higher risk of stroke than those without hemodynamic abnormalities. This suggests that the higher the SUA/Cr ratio and the more likely the individual is to have atherosclerosis, the lower the CVHI score will be.

### Limitations:

It includes small sample size, limited observation indexes, and short follow-up time. In future clinical studies, the sample size will be further expanded, more outcome and follow-up parameters will be added, and the outcome of high-risk individuals after drug intervention will be included to bring more benefits to patients.

## CONCLUSIONS

Stroke occurs increasingly every year, and conditions such as advanced age, poor lifestyle habits, and the presence of underlying diseases are risk factors for its onset. It is of great significance that scientific and effective monitoring, assessment and prevention should be carried out and relevant policies should be formulated for high-risk groups; the SUA/Cr ratio can be used to determine the risk factors of stroke, which is of clinical significance. For those with a high SUA/Cr ratio (>5.55), further education on stroke and other related knowledge should be strengthened, and regular and proper examination of CVHI should be performed in order to identify individuals at high risk of stroke as early as possible.

### Authors’ Contributions:

**XB** and **HW:** Carried out the studies, data collectdion, and drafted the manuscript, and are responsible and accountable for the accuracy or integrity of the work.

**JL** and **JX:** Performed the statistical analysis and participated in its design.

**PC:** Literature search**,** performed the statistical analysis , critical review and participated in its design.

All authors read and approved the final manuscript.
